# Clonal lineage and biofilm growth shape cefiderocol activity in *Acinetobacter baumannii* from oncology patients

**DOI:** 10.3389/fcimb.2026.1788718

**Published:** 2026-04-14

**Authors:** Ilaria Cavallo, Francesca Sivori, Mauro Truglio, Massimo Francalancia, Elva Abril, Giorgia Fabrizio, Sara Petrolo, Francesca Maione, Grazia Prignano, Arianna Mastrofrancesco, Fulvia Pimpinelli, Enea Gino Di Domenico

**Affiliations:** 1 Microbiology and Virology, San Gallicano Dermatological Institute, IRCCS, Rome, Italy; 2 Department of Biology and Biotechnology “C. Darwin”, Sapienza University of Rome, Rome, Italy

**Keywords:** *Acinetobacter baumannii*, avibactam, biofilm, carbapenem, cefiderocol, crab, β-lactamases

## Abstract

**Introduction:**

*Acinetobacter baumannii* is a leading cause of healthcare-associated infections in immunocompromised patients and frequently exhibits multidrug resistance. Cefiderocol, a siderophore cephalosporin, is among the few remaining therapeutic options for infections caused by carbapenem-resistant *A. baumannii* (CRAB); however, its activity may differ by clonal lineage and can be further compromised in the biofilm state. This study investigates genomic features and cefiderocol efficacy against planktonic and biofilm-associated forms of oncology-derived *A. baumannii* isolates.

**Methods:**

Twenty-five non-duplicate, consecutive clinical isolates of *A. baumannii* from oncology patients underwent whole-genome sequencing and multilocus sequence typing. Cefiderocol activity was quantified in planktonic and biofilm-associated states using minimum bactericidal concentration (MBC) and minimum biofilm eradication concentration (MBEC) assays.

**Results:**

Ten sequence types were identified, with the high-risk sequence type 2 (ST2) clone accounting for 56% (14/25) of isolates. ST2 strains showed significantly higher resistance to aminoglycosides, carbapenems, and fluoroquinolones than non-ST2 (NST) strains. The carbapenemase gene *bla*_OXA−23_ was detected exclusively in ST2. Colistin and cefiderocol were the most active agents overall. ST2 strains showed higher cefiderocol MBC values than NST strains. However, avibactam significantly reduced cefiderocol MBC in ST2, consistent with class D β-lactamases activity. ST2 and NST isolates exhibited comparable distributions of iron acquisition genes and similar CAS-detected siderophore activity under the assay conditions tested. Cefiderocol activity was significantly reduced in biofilms relative to planktonic cells (median MBEC 2 µg/ml versus median MBC 0.5 µg/ml). NST exhibited higher MBEC/MBC ratios than ST2 isolates, indicating greater biofilm-associated tolerance to cefiderocol.

**Discussion:**

Collectively, these data associate the predominance of oncology-derived ST2 with *bla*_OXA-23_ carriage and higher cefiderocol bactericidal thresholds and show that cefiderocol activity is consistently reduced in the biofilm state. Future studies integrating functional measures of iron acquisition and β-lactamase activity will be needed to define the determinants of cefiderocol efficacy across lineages and growth states.

## 1 Introduction

*Acinetobacter baumannii* is a Gram-negative opportunistic pathogen and a major cause of healthcare-associated infections, particularly in critically ill and immunocompromised patients (Cavallo et al., 2023). Its global spread and remarkable ability to acquire antibiotic resistance pose a serious challenge to infection management and patient outcomes, with mortality rates reaching up to 70% in severe infections such as ventilator-associated pneumonia or bacteremia (Lee et al., 2014; Boutzoukas and Doi, 2025). This pathogen exhibits a high capacity for developing resistance to multiple antibiotic classes, including β-lactams, aminoglycosides, and fluoroquinolones, primarily through horizontal gene transfer and adaptive resistance mechanisms (Peleg et al., 2008; Piperaki et al., 2019). Among resistant isolates, carbapenem-resistant *A. baumannii* (CRAB) has emerged as a critical nosocomial threat, frequently associated with ventilator-associated pneumonia, bloodstream infections, and urinary tract infections. The widespread dissemination of CRAB isolates, reported in up to 70% of cases, has severely limited effective treatment options, making it a priority for infection control and antimicrobial stewardship programs (Giammanco et al., 2017; Baleivanualala et al., 2023; Uskudar-Guclu et al., 2024). In Italy, the burden of CRAB remains among the highest in Europe. Recent surveillance data indicate that 86.7% of *A. baumannii* isolates recovered from hospitalized patients exhibit carbapenem resistance (Scaglione et al., 2025), with rates peaking at 100% during the COVID-19 pandemic and remaining persistently high thereafter (Petazzoni et al., 2023).

*A. baumannii* exhibits remarkable environmental persistence, enabling its widespread dissemination in healthcare settings and contributing to nosocomial outbreaks (Whiteway et al., 2022; Lucidi et al., 2024). A key determinant of its pathogenicity is its ability to form biofilms, which facilitate adherence to medical devices such as catheters and endotracheal tubes, leading to persistent infections (Greene et al., 2016). Biofilm formation dramatically reduces antibiotic susceptibility by providing a physical and metabolic barrier that shields bacterial cells from antimicrobial agents and host immune responses (Roy et al., 2022; Mendes et al., 2023). The OXA-_23_–producing *A. baumannii* sequence type 2 (ST2) lineage, classified as a high-risk clone, is broadly disseminated worldwide and has been documented in numerous countries across multiple continents (Hamidian and Nigro, 2019; Nghiem et al., 2025). These isolates are frequently recovered from intensive care units and are consistently recognized as a significant public health threat. Their tendency to acquire and accumulate antimicrobial resistance, along with their substantial pathogenic potential, has raised concerns among clinicians and researchers (Wei et al., 2023; Hummel et al., 2024; Brek et al., 2025). Furthermore, biofilm-associated growth enhances horizontal gene transfer, accelerating the dissemination of antimicrobial resistance elements. In ventilator-associated pneumonia, biofilms have been shown to promote the exchange of carbapenemase-encoding genes, such as *bla*_OXA-23_ and *bla*_NDM_, further complicating treatment and contributing to the persistence of CRAB in clinical settings (Roy et al., 2022; Sharma et al., 2023). In catheter-associated bloodstream infections, multidrug-resistant (MDR) *A. baumannii* isolates frequently form biofilms with increased biomass and enhanced tolerance to last-line antibiotics, including colistin and tigecycline, complicating treatment strategies (Rodríguez-Baño et al., 2008; Sato et al., 2021). Moreover, *A. baumannii* biofilms are implicated in skin and soft tissue infections, contributing to disease persistence and chronicity (Albrecht et al., 2006; Kasperski et al., 2023). Collectively, this biofilm-mediated tolerance contributes substantially to persistence and treatment failure (Cavallo et al., 2023; Mendes et al., 2023). Given this complex resistance ecology, cefiderocol, a novel siderophore cephalosporin, represents an emerging option against carbapenem-resistant Gram-negative pathogens, including *A. baumannii* (Karruli et al., 2023). Its unique mechanism of action exploits bacterial iron uptake systems to facilitate intracellular drug delivery, bypassing common resistance mechanisms such as porin loss and efflux pumps (Artuso et al., 2023; Kaye et al., 2023). Notably, cefiderocol exhibits stability against carbapenemases and potent *in vitro* and *in vivo* activity against CRAB, making it a valuable alternative to colistin-based regimens (Falcone et al., 2022). However, evidence regarding the efficacy against biofilm-associated *A. baumannii* infections remains limited. Preliminary studies suggest that the biofilm structure and density may influence cefiderocol’s penetration and activity, underscoring the need for further investigation (Yousefi et al., 2024).

This study evaluates the effectiveness of cefiderocol against clinical *A. baumannii* isolates obtained from oncology patients. Due to immunosuppression and the frequent use of invasive devices, this patient population is particularly vulnerable to biofilm-related infections. By focusing on biofilm phenotypes and resistance profiles, this work aims to elucidate the therapeutic potential of cefiderocol and contribute to the development of more targeted strategies to combat biofilm-mediated resistance in this high-risk cohort.

## 2 Methods

### 2.1 Strain collection and ethical approval

This study was conducted at the San Gallicano Dermatological Institute and the Regina Elena National Cancer Institute from September 2023 to December 2024. A total of twenty-five non-duplicate, consecutive clinical strains of *A. baumannii* were isolated from adult patients with hematological malignancies and solid tumors hospitalized at the Regina Elena National Cancer Institute. This study received approval from the Central Ethics Committee of I.R.C.C.S. Lazio (Prot. 1716/22 — 23.08.2022, N. 1716/22), and all procedures were in accordance with established ethical guidelines and local regulatory requirements.

### 2.2 Patient enrollment and bacterial identification

Clinical samples for microbiological analysis were collected by ward nurses and transported to the Microbiology and Virology Laboratory at the San Gallicano Institute. A total of 25 A*. baumannii* isolates were recovered from clinical samples collected from 11 female and 14 male patients. Samples included blood (n = 5), bronchoalveolar lavage (n = 11), sputum (n = 2), and skin swabs (n = 7). Bacterial identification was performed by MALDI-TOF MS (Bruker Daltonik, Bremen, Germany). Antibiotic susceptibility testing was conducted using the automated BD Phoenix™ system (Becton Dickinson Diagnostic Systems, Sparks, MD, USA). Colistin susceptibility was further determined by broth microdilution tests performed according to EUCAST reference guidelines. Results were interpreted using EUCAST breakpoints (www.eucast.org). Isolates were classified according to the European Centre for Disease Prevention and Control (ECDC) definitions for antimicrobial resistance profiles (https://www.ecdc.europa.eu/en).

The multiple antibiotic resistance (MAR) index was calculated for each isolate using the formula MAR Index = a/b, where “a” denotes the number of antibiotics to which the isolate was resistant and “b” the total number of antibiotics tested (Krumperman, 1983).

### 2.3 Cefiderocol minimum inhibitory concentration and minimum bactericidal concentration

Antimicrobial susceptibility was assessed using the reference broth microdilution method. Cefiderocol testing was performed in Chelex-treated, iron-depleted, cation-adjusted Mueller–Hinton broth (ID-CAMHB) across a concentration range of 0.008–128 µg/ml. Bacterial suspensions without antibiotic exposure served as growth controls. Details regarding Chelex treatment, iron measurement, quality control procedures, and microdilution panel preparation have been described previously (Hackel et al., 2018). MICs for cefiderocol were interpreted according to EUCAST clinical breakpoints, and MICs ≤ 2 µg/ml were classified as susceptible (Pasteran et al., 2024). Each isolate was tested in duplicate, and MIC values were consistent across replicates in all cases. For MBC determination, aliquots from wells at and above the MIC were plated after incubation, and viable counts were expressed as CFU/ml. The MBC was defined as the lowest cefiderocol concentration producing a ≥3 log10 reduction in CFU/ml relative to the antibiotic-free growth control (DeJonge et al., 2025; Fabrizio et al., 2025).

### 2.4 Cefiderocol minimum biofilm eradication concentration assays

An overnight culture of *A. baumannii* grown on MacConkey agar was used to inoculate 2 ml of 0.45% saline to a 0.5 McFarland turbidity standard (~10^8^ CFU/ml). Diluted cell suspensions (~10^5^ CFU/ml) were added to a 96-well polystyrene plate containing 100 µL of ID-CAMHB for biofilm formation. After 5 hours of incubation at 37 °C, the wells were rinsed with 0.45% saline to remove non-adherent bacteria, and the biofilm cells were resuspended in 100 µL of ID-CAMHB containing serial dilutions of cefiderocol (0.008 to 128 µg/ml). The plate was incubated for an additional 20 hours at 37 °C. Following antibiotic exposure, the well contents were collected and rinsed twice with sterile PBS, and the biofilms were resuspended in 100 µL of 0.45% saline (Peeters et al., 2009). Biofilm cells were scraped and serially diluted for enumeration of CFUs on MacConkey agar. MBEC was the lowest antibiotic concentration that resulted in a ≥3 log10 reduction in CFU/ml of biofilm-embedded bacteria compared with the untreated control. The MBEC/MBC ratio was calculated to assess biofilm tolerance, indicating the fold increase in the antibiotic dosage required to inhibit or kill biofilm-associated cells compared to planktonic cells (Sivori et al., 2024b).

### 2.5 Cefiderocol and avibactam synergy testing

The potential synergistic activity of cefiderocol with avibactam (4 µg/ml) was assessed using the broth microdilution method in ID-CAMHB, as described previously (Abdul-Mutakabbir et al., 2021; Satlin et al., 2023). A bacterial suspension (~10^5^ CFU/ml) without antibiotics served as a growth control. Details on chelation, iron concentration determination, quality control, and broth microdilution preparation have been previously reported (Hackel et al., 2018). MBC was determined as previously described (Sivori et al., 2024b).

### 2.6 Quantitative measurement of siderophore production

For each bacterial culture, 500 µL of ID-CAMHB, prepared with Chelex^®^ treatment (Bio-Rad, Hercules, CA, USA), was inoculated with 5 µL of bacterial suspension (~1 × 10^5^ CFU/ml). After 48 hours of static incubation at 37 °C, bacterial cultures were centrifuged, and supernatants were collected to estimate siderophore production. Siderophore activity was measured using the Chrome Azurol S (CAS) assay (Guembe et al., 2025). Absorbance at 630 nm was measured using a Multiskan SkyHigh (Thermo Fisher Scientific, USA). Using Payne’s formula, siderophore production was expressed as percent siderophore units (PSU) (Payne, 1993).

### 2.7 Biofilm biomass quantification, viable cell counts, and extracellular DNA quantification

Biofilm formation was further evaluated using crystal violet staining in sterile polystyrene 96-well plates. Early biofilm biomass was measured after 5 hours by removing non-adherent cells and gently washing wells with 0.45% saline before crystal violet staining. Biofilm biomass was also measured after 24 h incubation at 37 °C and quantified as described previously (Sivori et al., 2024a). Viable cell counts were assessed using plate enumeration to determine CFU/ml. All experiments were conducted in triplicate and repeated three times. Biofilm eDNA was quantified using a PicoGreen fluorescence assay, adapted from a published protocol (Fabrizio et al., 2025).

### 2.8 Whole genome sequencing and typing

Genomic DNA was extracted from *A. baumannii* isolates using the QIAamp DNA Mini Kit (QIAGEN, Hilden, Germany) according to the manufacturer’s instructions. DNA quality was assessed by 0.8% agarose gel electrophoresis and quantified with a NanoDrop 2000 spectrophotometer (Thermo Fisher, Wilmington, DE, USA). Whole-genome sequencing was performed using Illumina’s massive parallel sequencing (MPS) technology. High-quality reads were assembled into scaffolds and annotated using the Bactopia suite v. 3.1.0.

Multilocus sequence typing (MLST) was performed by comparing genome assemblies to the PubMLST database for *A. baumannii*. Each unique allele combination corresponded to a specific sequence type (ST). The Pasteur MLST scheme, targeting seven housekeeping genes (*cpn60, fusA, gltA, pyrG, recA, rplB*, and *rpoB*), was used, as it enables the identification of distantly related clonal lineages despite its lower discriminatory power compared to the Oxford scheme (Gaiarsa et al., 2019). Sequence analysis was performed by uploading FASTA files to the *A. baumannii* typing database (https://pubmlst.org/bigsdb?db=pubmlst_abaumannii_seqdef).

### 2.9 Genotypic characterization of *A. baumannii*

Antibiotic resistance genes were identified using the Comprehensive Antibiotic Resistance Database (CARD) (https://card.mcmaster.ca/home). FASTA sequences were uploaded to the Resistance Gene Identifier (https://card.mcmaster.ca/analyze/rgi), which predicts resistomes based on homology and SNP models, applying strict filtering criteria for high-quality genome coverage.

Virulence factors were identified using the Virulence Factor Database (VFDB) (http://www.mgc.ac.cn/VFs/main.htm). Only matches with ≥80% coverage and ≥90% identity were considered. Data were processed using a custom Python script (Pandas, Conda) to convert JSON outputs into CSV files and create presence-absence data frames.

To investigate the role of iron in modulating virulence and resistance, we employed FeGenie (https://github.com/Arkadiy-Garber/FeGenie), which detects genes involved in iron acquisition, storage, and cycling. FASTA files were processed to generate CSV files summarizing gene presence and iron-related genomic features. COG categories and their abundances were computed using eggNOG-mapper (Cantalapiedra et al., 2021) v.2.1.12 against the eggNOG database (Huerta-Cepas et al., 2017) v.5.0, with default parameters. To test for differences in the distribution of COG functional categories between the two sample groups, a contingency table of category counts was constructed and group-level differences were assessed using a Pearson chi-square test. Standardized Pearson residuals were then examined to identify categories contributing most to the overall association; categories with residuals > 2 or < −2 were considered over- or under-represented in one group relative to the expected counts under independence.

### 2.10 Biofilm imaging

Biofilms were established in μ-Slides (Ibidi, Gräfelfing, Germany) by inoculating each slide with approximately 1 × 10^5^ cells suspended in 500 μL of fresh ID-CAMHB medium, followed by incubation at 37 °C for 24 hours. Biofilms were stained using the LIVE/DEAD BacLight Bacterial Viability Kit (Life Technologies, New York, NY, United States) following the manufacturer’s instructions. Stained biofilms were then examined using an Axio Observer inverted fluorescence microscope equipped with an Apotome system (Carl Zeiss International, Oberkochen, Germany). Image analysis was performed with ZEN 3.2 (blue edition) software, as previously described (Fabrizio et al., 2024).

### 2.11 Statistical analysis

Variables were summarized using descriptive statistics. Continuous variables were compared using Student’s t-test or the Mann-Whitney U test, depending on data distribution. Categorical variables were analyzed with χ^2^ or Fisher’s exact test. Correlation analysis was performed using Spearman’s rank correlation coefficient (ρ). A P-value <0.05 was considered statistically significant. All statistical analyses were conducted using SPSS version 21 (SPSS Inc., Chicago, IL, USA).

## 3 Results

### 3.1 Phylogenetic analysis and genomic characterization

**Figure 1:**
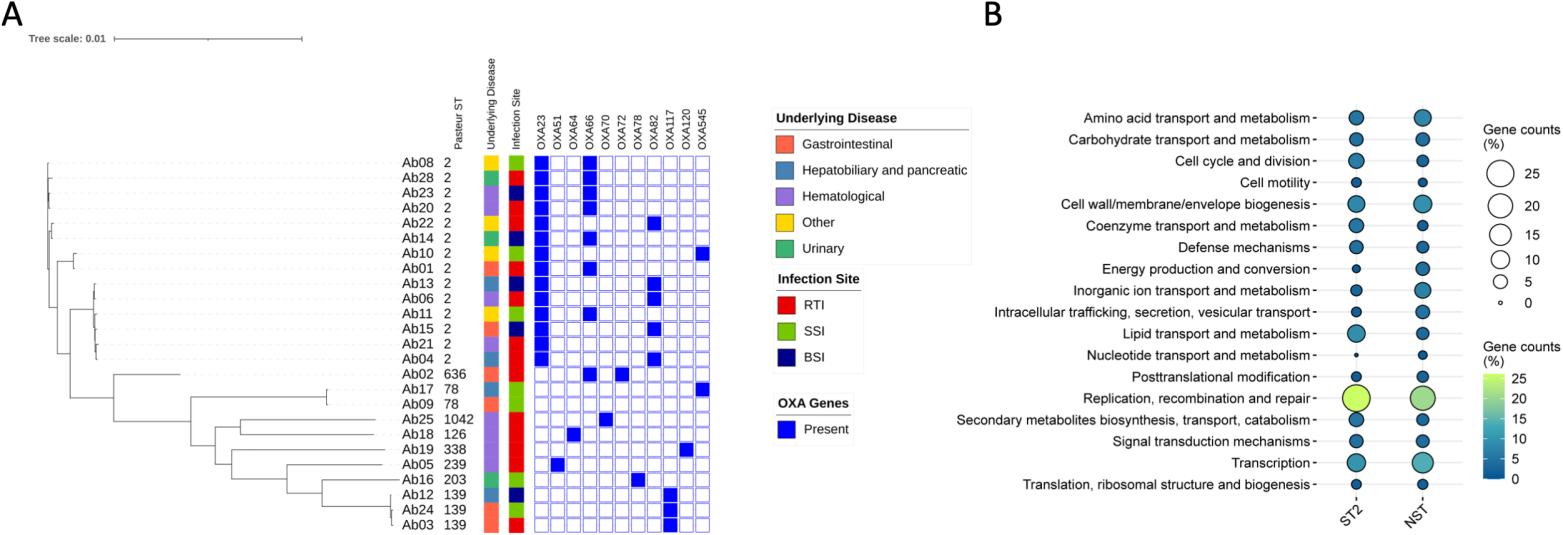
Maximum-likelihood phylogeny of clinical *A. baumannii* isolates based on whole-genome SNP alignment. **(A)** Metadata includes the patient’s underlying disease (color-coded), the infection site (RTI, respiratory tract infection; SSI, surgical site infection; BSI, bloodstream infection), and the presence of OXA-type β-lactamase genes (blue boxes). Isolates were clustered based on genetic similarity and annotated for clinical context and resistance genotype. The *A. baumannii* reference strain ATCC 19606 (ST46) was used as a reference genome. **(B)** Functional category analysis of Clusters of Orthologous Genes (COGs) based on unique genes identified in each group. Values for each group indicate the absolute number of identified COGs.

Between September 2023 and December 2024, twenty-five *A. baumannii* strains were collected from oncology patients with hematological malignancies and solid tumors. Genotyping of the isolates was performed using MLST based on the pubMLST database, employing the Pasteur scheme. Ten distinct STs were identified. ST2 was the most prevalent, occurring in 14/25 samples (56%), followed by ST139 in 3/25 samples and ST78 in 2/25 samples. ST126, ST203, ST239, ST338, ST636, and ST1042 were each detected in a single sample. No clonal strains were detected; all isolates displayed distinct core-genome profiles. ASA^3^P generated a phylogenetic tree (**Figure 1A**) using consensus sequences derived from single-nucleotide polymorphisms (SNPs). All ST2 isolates clustered into a single clade, clearly separated from the other STs. Accordingly, the isolates were grouped into two main clusters: ST2 and non-ST2 (NST). The *A. baumannii* reference genome provided by the NCBI database (ATCC 19606, belonging to ST46) was included in the analysis. ASA^3^P estimated a pangenome comprising 12,278 genes. Genomic analysis of the *A. baumannii* isolates revealed 1,930 core genes (15.7%), 4,977 accessory genes (40.5%), and 5,371 singletons (43.8%), genes found in only one genome within the analyzed set. The *bla*_OXA-23_ gene, a major determinant of carbapenem resistance, was detected exclusively in ST2 isolates and was absent in all NST strains. No significant association was observed between genetic clustering and infection site or underlying disease, indicating that host clinical factors did not drive phylogenetic structure. Functional profiling of annotated singleton genes revealed a significant shift in COG category distribution between ST2 and NST isolates (χ^2^ = 51.21, P < 0.0001). Specifically, ST2 was enriched for genes assigned to cell cycle and division, coenzyme transport and metabolism, and lipid transport and metabolism, whereas NST showed relative enrichment for genes involved in energy production and conversion, inorganic ion transport and metabolism, and intracellular trafficking, secretion, and vesicular transport (**Figure 1B**).

### 3.2 Characterization of resistance genes and antibiotic susceptibility profiles

**Figure 2:**
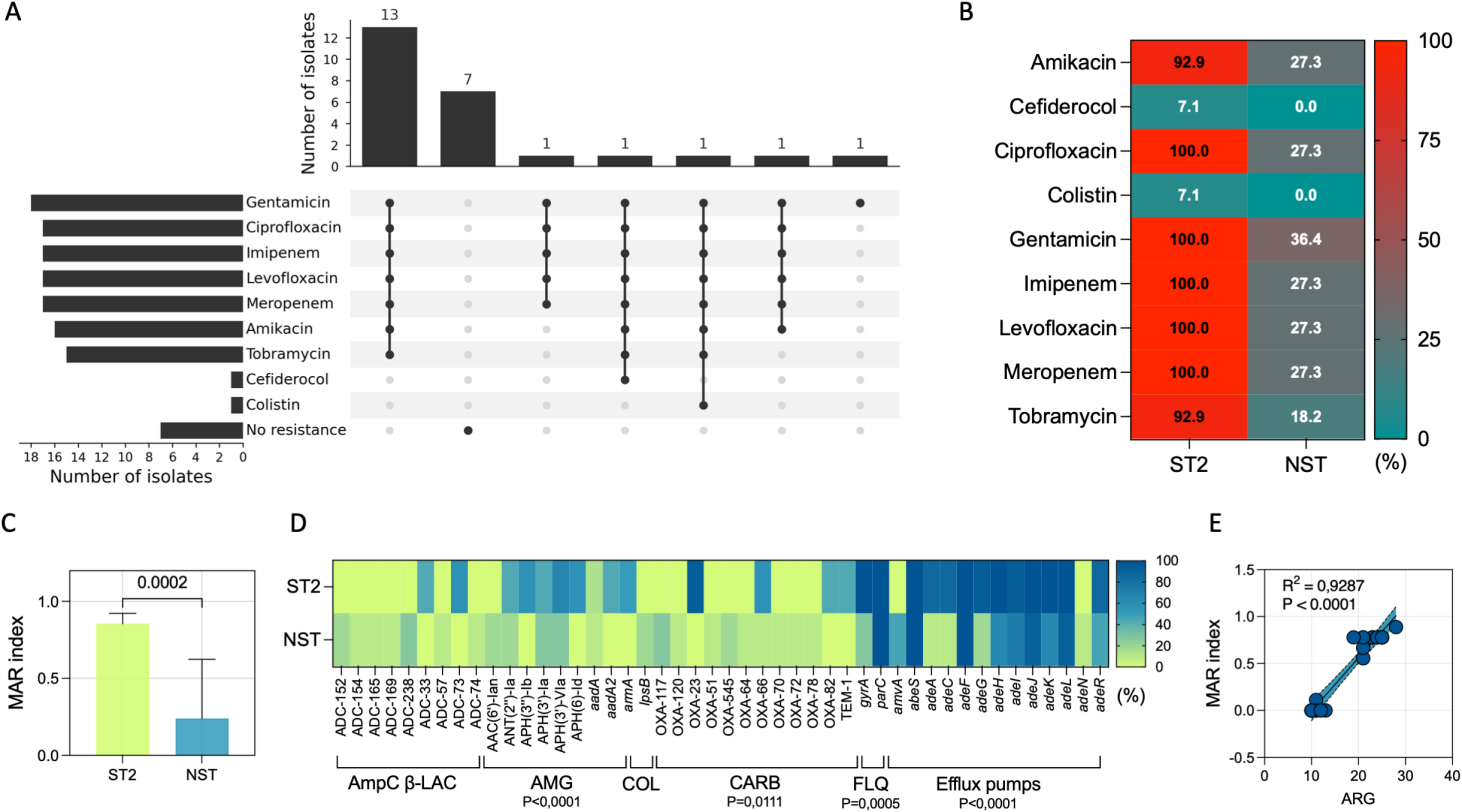
Genotypic characterization of antimicrobial resistance profiles in *A. baumannii* isolates. **(A)** UpSet plot summarizing combinations of phenotypic resistance across the tested antibiotics. Horizontal bars indicate the number of isolates resistant to each agent; vertical bars indicate the number of isolates sharing each resistance combination (filled circles). **(B)** Heat map showing the proportion (%) of resistant isolates within ST2 (n = 14) and NST (n = 11) for each antibiotic; values are overlaid within tiles. **(C)** Multiple antibiotic resistance (MAR) index by lineage; center values and error bars denote mean ± standard deviation; P value indicates the between-group comparison (Mann–Whitney U test). **(D)** Heat map of antimicrobial resistance gene (ARG) prevalence by lineage, expressed as the percentage of isolates carrying each determinant (color scale). Determinants are grouped by functional class (AmpC β-lactamases, aminoglycoside resistance, colistin-associated loci, carbapenemases/β-lactamases, fluoroquinolone resistance determinants, and efflux pumps). P values beneath brackets indicate between-group differences in class-level ARG burden (χ^2^ test). **(E)**, Linear regression between total ARG count and MAR index across isolates; R^2^ and P value are shown.

Phenotypic susceptibility testing revealed heterogeneous resistance patterns across the cohort (**Figure 2**). Altogether, seven unique AMR profiles, each with different combinations of resistance phenotypes, were detected (**Figure 2A**). The dominant phenotype comprised concurrent resistance to aminoglycosides, fluoroquinolones, and carbapenems (n = 13), whereas a substantial fraction of isolates showed no resistance to the tested panel (n = 7). Stratification by lineage highlighted a marked enrichment of antibiotic resistance in the high-risk ST2 group compared to NST (**Figure 2B**). ST2 isolates exhibited significantly higher resistance to aminoglycosides, specifically amikacin (P = 0.0007), gentamicin (P < 0.0001), and tobramycin (P = 0.0001), compared to NST isolates. Furthermore, ST2 isolates demonstrated significantly higher resistance to imipenem (P < 0.0001), meropenem (P < 0.0001), and fluoroquinolones (P < 0.0001) than NST isolates. By contrast, only one isolate was resistant to colistin (Ab14), and only one was non-susceptible to cefiderocol (Ab13). Consistent with the cumulative resistance burden, the multiple antibiotic resistance (MAR) index was significantly higher in ST2 than NST (P = 0.0002) (**Figure 2C**).

Whole-genome sequencing resolved the genetic basis of these phenotypes and indicated strong genotype–phenotype concordance (**Figures 2D, E**). ST2 isolates carried a significantly higher burden of antimicrobial resistance genes (ARG) than NST isolates (P < 0.0001), with enrichment of genes associated with aminoglycoside resistance (P < 0.0001), carbapenem resistance (P = 0.0111), fluoroquinolone resistance (P = 0.0005) and efflux systems (P < 0.0001) (**Figure 2D**). The carbapenemase gene *bla*_OXA-23_ in association with the *bla*_OXA-51_-like variants (*bla*_OXA-66_ or *bla*_OXA-82_ genes) detected in ST2, was consistent with the predominance of CRAB in this lineage. Across all isolates, the MAR index correlated strongly with the number of detected ARGs (R^2^ = 0.9287, P < 0.0001) (**Figure 2E**), supporting a quantitative relationship between resistome size and phenotypic multidrug resistance.

### 3.3 Cefiderocol efficacy against planktonic *A. baumannii*

**Figure 3:**
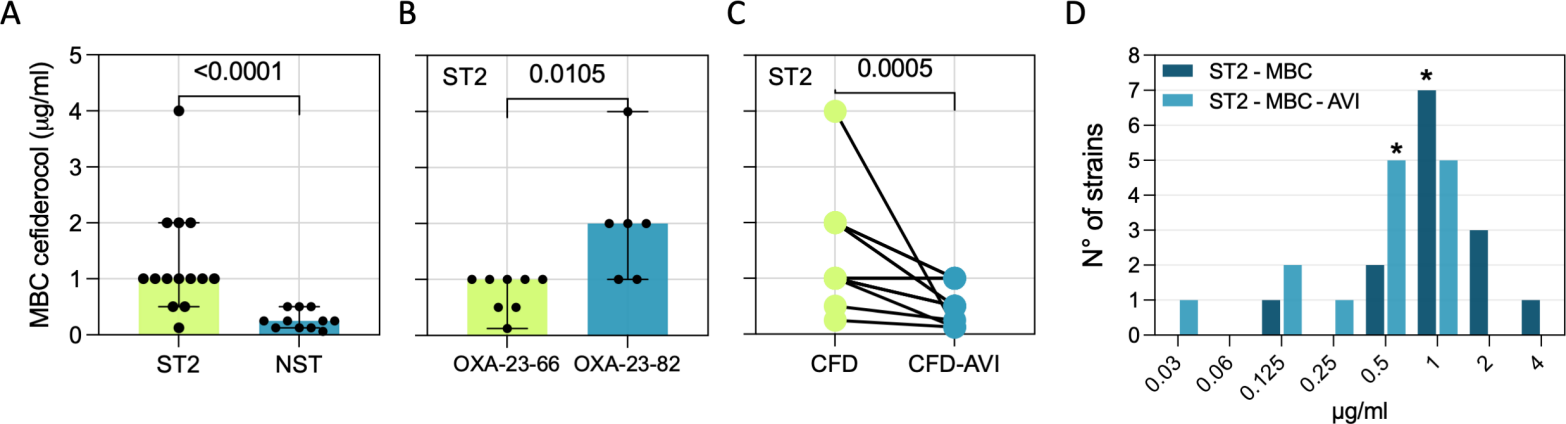
Cefiderocol susceptibility. **(A)** Box plot representing the MBC values stratified between the ST2 and NST groups. **(B)** Box plot of MBC for cefiderocol comparing *A. baumannii* strains harboring the *bla*_OXA-23_ and *bla*_OXA-66_ genes with those harboring *bla*_OXA-23_ and *bla*_OXA-82_. **(C)** Comparison of MBC values between cefiderocol alone and cefiderocol with avibactam (AVI). **(D)** MBC values for cefiderocol and the synergistic effect between cefiderocol and AVI. * above the bars indicates the median values.

To complement MIC-based susceptibility data, which capture growth inhibition but not killing, we determined cefiderocol MBC as a quantitative measure of bactericidal activity. Notably, MBC​ values were completely concordant with MIC values across all isolates, with no MIC–MBC​ discordance observed. Given the higher multidrug-resistance burden in ST2 and its consistent carriage of *bla*_OXA-23_ with *bla*_OXA-66_ or *bla*_OXA-82_ genes, we evaluated whether cefiderocol MBC differed between ST2 and NST isolates. As shown in **Figure 3A**, the MBC values for cefiderocol were significantly higher (P < 0.0001) in ST2 (median 1 mg/L; range 0.125–4) strains than in NST strains (median 0.25 mg/L; range 0.064–0.5). Within the ST2 group, strains co-harboring *bla*_OXA-23_ and *bla*_OXA-82_ showed a median MBC of 2 mg/L (1–4), which was significantly higher (P = 0.0105) than that observed in strains carrying *bla*_OXA-23_ *bla*_OXA-66_, with a median MBC of 1 mg/L (0.125–1) (**Figure 3B**). To assess the direct role of β-lactamases, cefiderocol MBC values were measured in the presence of the β-lactamase inhibitor avibactam in ST2 strains positive for *bla*_OXA-23_ (**Figure 3C**). All strains showed a significant reduction (P = 0.0005) in cefiderocol MBC when treated with avibactam. In particular, Ab13, the only cefiderocol-non-susceptible isolate with MBC = 4 μg/ml, showed a marked reduction in cefiderocol MBC_9_ to 0.125 μg/ml in the presence of avibactam, consistent with a substantial β-lactamase contribution to reduced cefiderocol killing in this strain. Notably, the median MBC value for cefiderocol decreased from 1 μg/ml to 0.5 μg/ml in the presence of avibactam (**Figure 3D**).

### 3.4 Distribution of iron-related genes and siderophore production

**Figure 4:**
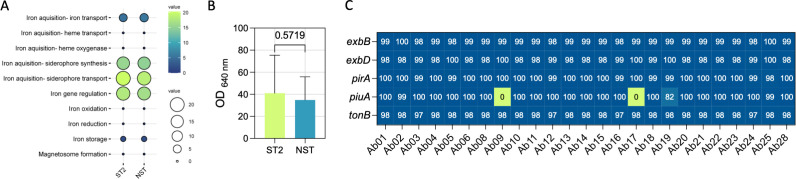
Presence of iron metabolism-related genes and siderophore production. **(A)** Distribution of iron-related gene categories in the ST2 and NST groups; **(B)** Measurement of siderophore production in the ST2 and NST groups. Siderophore production was expressed as percent siderophore units (PSU); **(C)** Presence (blue), absence (green), and sequence homology of siderophore production genes across different isolates.

Given that cefiderocol activity may be influenced by iron metabolism, iron-related gene content and CAS-detected siderophore activity were compared between ST2 and NST isolates. Genes involved in siderophore biosynthesis, transport, and regulation were similarly distributed across the two groups (**Figure 4A**), and no significant difference in siderophore activity was detected under the assay conditions used (**Figure 4B**). In parallel, key iron acquisition and homeostasis loci potentially relevant to cefiderocol susceptibility were examined for gene absence or sequence variation (**Figure 4C**). Two NST strains (Ab09 and Ab17) lacked *piuA*, whereas no variants were identified in *exbB*, *exbD*, *pirA*, or tonB among the remaining isolates, including the cefiderocol-non-susceptible strain Ab13. Taken together, these findings do not indicate major differences in iron-related gene content within the loci examined. Nevertheless, because the transcriptional and proteomic regulation of iron uptake systems was not assessed, differential expression of these determinants as contributors to cefiderocol susceptibility, particularly under biofilm conditions, cannot be excluded.

### 3.5 Biofilm characterization

**Figure 5:**
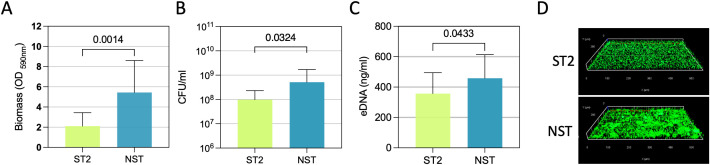
Biofilm characterization. **(A)** Total biomass assessed by crystal violet staining. **(B)** Viable cell counts (CFU/ml). **(C)** extracellular DNA (eDNA) content. Data are presented as mean ± SD, with statistical significance indicated. **(D)** Fluorescence microscopy images of *A. baumannii* biofilms stained with the live/dead assay. Live cells are stained with SYTO9 (green fluorescence), while dead cells are stained with propidium iodide (red fluorescence). Images are 3D reconstructions from z-stack acquisitions. Scale bars are indicated.

Each *A. baumannii* strain was characterized in ID-CAMHB for biomass production, biofilm cell count (CFU/ml), and eDNA (**Figures 5A–C**). NST strains demonstrated significantly greater efficiency in biomass production (P = 0.0014), biofilm cell count (P = 0.0324), and eDNA (P = 0.0433) compared to ST2 strains. Microscopic analysis of *A. baumannii* biofilm morphology revealed uniform biofilm formation after 24 hours, characterized by cellular aggregates and structures exceeding 10 μm in height (z-projection). Consistent with the plate assay results, NST strains generally exhibited higher biomass production and thickness than ST2 strains (**Figure 5D**).

### 3.6 The role of biofilm in cefiderocol tolerance

**Figure 6:**
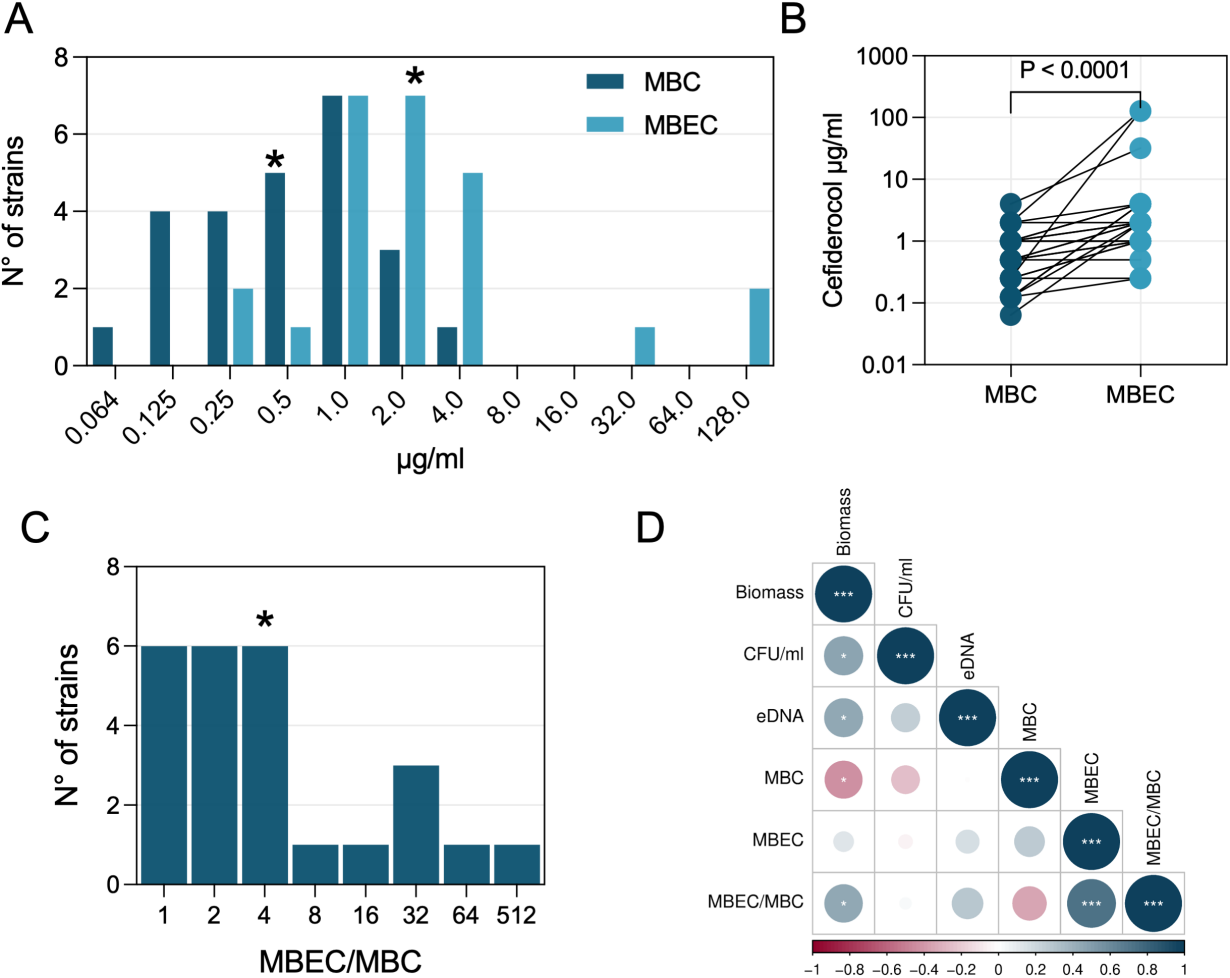
Growth-state effects on cefiderocol activity. **(A)** Frequency distributions of cefiderocol minimum bactericidal concentrations for planktonic cells (MBC, dark bars) and minimum biofilm eradication concentrations (MBEC, light bars) across *A. baumannii* clinical isolates (n = 25). **(B)** Paired comparison of MBC and MBEC for individual isolates; lines connect matched measurements. P-value denotes the paired statistical comparison (two-sided Wilcoxon matched-pairs signed-rank test). **(C)** Distribution of MBEC/MBC ratios, indicating the fold increase in cefiderocol required to eradicate biofilm-embedded cells relative to planktonic cells. Asterisks indicate median values. **(D)** Spearman’s rank correlation matrix illustrates the relationships among biomass, viable cells (CFU/ml), eDNA content, MBC, MBEC, and MBEC/MBC ratio. Statistical significance of Spearman correlations is indicated by **∗**P < 0.05, **∗∗**P < 0.01, and **∗∗∗**P < 0.001.

Given the significantly greater biofilm biomass, viable biofilm burden, and eDNA content observed in NST isolates relative to ST2, we tested whether biofilm-associated growth differentially impacts cefiderocol bactericidal activity across sequence types. Cefiderocol activity was quantified for planktonic and biofilm-embedded cells by determining MBC and MBEC, respectively (**Figure 6**; **Table 1**). Across the isolates, biofilm growth was associated with a median MBEC of 2 μg/ml (range: 0.25–128 μg/ml), which was significantly higher (P < 0.0001) than the median MBC of 0.5 μg/ml (range: 0.064–4 μg/ml) (**Figures 6A, B**). The MBEC/MBC ratio, representing the increase in antimicrobial concentration required to inhibit biofilm-associated cells relative to planktonic cells, had a median of 4-fold, with values ranging from 1-fold to 512-fold across isolates. The MBEC/MBC ratio varied significantly between STs. NST isolates, which formed denser and more structured biofilms, exhibited significantly higher MBEC/MBC ratios compared to ST2 isolates (P = 0.0130), suggesting a greater degree of biofilm-associated cefiderocol tolerance (**Table 1**; **Figure 6C**).

**Table 1:** Minimum bactericidal concentration (MBC), minimum biofilm eradication concentration (MBEC), and MBEC/MBC ratio of cefiderocol against ST2 and non-ST2 (NST) *A. baumannii* isolates.

Sequence type	MBC (μg/ml)	MBEC (μg/ml)	MBEC/MBC
ST2	1 (0.125 – 4)	2 (0.50 – 128)	2 (1 – 64)
NST	0.25 (0.064 - 0.5)	2 (0.25 – 128)	4 (1 – 512)
P value	< 0.0001	ns (0.4319)	0.013

MBC values indicate the median (range) cefiderocol concentration (μg/ml) required to achieve bactericidal activity in planktonic cells, whereas MBEC values reflect the median (range) cefiderocol concentration (μg/ml) required to eradicate biofilm-embedded cells. The MBEC/MBC ratio quantifies the fold increase in cefiderocol dosage required to eradicate biofilm compared to the planktonic state. Statistical comparisons between ST2 and NST groups were performed using the Mann-Whitney test.

To further explore the relationships between biofilm-related phenotypes and cefiderocol tolerance, a correlation matrix analysis was performed integrating biofilm biomass, viable biofilm cells (CFU/ml), eDNA content, MBC, MBEC, and the MBEC/MBC ratio (**Figure 6D**). Biofilm biomass showed a significant positive correlation with both viable biofilm cells (P = 0.0197) and eDNA content (P = 0.0219), indicating that isolates forming larger biofilms also accumulated greater amounts of extracellular matrix components. In addition, biofilm biomass was inversely correlated with MBC (P = 0.0307) and positively correlated with the MBEC/MBC ratio (P = 0.0256). indicating that higher biomass was associated with increased biofilm-associated cefiderocol tolerance. Importantly, biomass quantified after 5 hours strongly correlated with the corresponding 24-hour biomass values across isolates (**Supplementary Figure 1**), indicating that inter-isolate differences in biofilm-forming capacity were already evident at the time MBEC testing was initiated.

## 4 Discussion

The study revealed substantial genetic heterogeneity among *A. baumannii* isolates, identifying ten distinct STs, with ST2 as the predominant lineage. This finding aligns with previous reports highlighting ST2 as the most globally disseminated clone, frequently associated with MDR, hospital outbreaks, and poor clinical outcomes (Peleg et al., 2008; Karampatakis et al., 2017; Valcek et al., 2023). The widespread prevalence of ST2 is likely driven by its enhanced resistance and virulence traits, which promote persistence, survival, and transmission in healthcare settings, particularly among immunocompromised patients (Zhang et al., 2021; Cavallo et al., 2023).

From both phylogenetic and clinical perspectives, the dominance of ST2 in 56% of isolates is concerning, as this lineage exhibited significantly higher antimicrobial resistance than NST. The study highlights the high burden of antibiotic-resistant isolates, with 68% classified as CRAB. Notably, all ST2 isolates were carbapenem-resistant and carried a broad repertoire of aminoglycoside and fluoroquinolone-resistance genes, further complicating treatment options.

A key resistance determinant in these isolates was *bla*_OXA-23_, encoding a class D β-lactamase responsible for carbapenem resistance. None of the strains harbored *bla*_NDM_ or other metallo-β-lactamase (MBL) genes. This distribution is consistent with the epidemiology reported in Italy, where OXA-type carbapenemases predominate among CRAB isolates. In a multicenter Italian study including 141 CRAB isolates, *bla*_NDM_ was not detected, further supporting the predominance of OXA-type resistance mechanisms in this setting (Segatore et al., 2022). Similarly, in a more recent Italian study of 110 consecutive CRAB bloodstream isolates collected between 2021 and 2023, all resistant isolates carried *bla*_OXA-23_, while only three isolates also harbored *bla*_NDM-1_. WGS showed close clonal relatedness among the *bla*_NDM-1_-positive isolates (ST231), suggesting localized outbreak dynamics rather than widespread national dissemination (Bianco et al., 2026).

The global dissemination of *bla*_OXA-23_-positive CRAB strains, frequently associated with ST2, has been widely documented, underscoring their role in healthcare-associated outbreaks and treatment failures (Mugnier et al., 2009). Given its clinical impact, *bla*_OXA-23_ represents a critical target for infection control and antimicrobial stewardship strategies.

In contrast, *bla*_OXA-66_, a variant of the *bla*_OXA-51_ β-lactamase, is not inherently associated with carbapenem resistance but may contribute to a highly resistant phenotype when co-expressed with other resistance mechanisms, such as overexpression or the presence of additional carbapenemase genes (Cherubini et al., 2022). While less efficient than *bla*_OXA-23_, *bla*_OXA-66_ remains a relevant marker in MDR *A. baumannii* infections. Our findings confirmed that all ST2 isolates carried *bla*_OXA-23_ in combination with either *bla*_OXA-66_ or *bla*_OXA-82_. The co-existence of multiple class D β-lactamase genes is particularly concerning, as bla_OXA-23_ serves as the primary carbapenemase while *bla*_OXA-66_ and *bla*_OXA-82_, both variants of *bla*_OXA-51_, may contribute to a complex resistance phenotype. This interplay likely exacerbates β-lactam resistance, making these infections particularly difficult to treat, reinforcing the urgent need for enhanced molecular surveillance. Among emerging treatments, cefiderocol has demonstrated *in vitro* activity against most β-lactamases and has been approved by the Food and Drug Administration (FDA) and European Medicines Agency (EMA) for the treatment of complicated Gram-negative infections (Kimbrough et al., 2024). Cefiderocol retained *in vitro* activity against nearly all isolates, with a single non-susceptible strain, supporting its potential utility against multidrug-resistant *A. baumannii* in this cohort. These results align with previous large-scale analyses reporting potent *in vitro* activity of cefiderocol against multidrug-resistant *A. baumannii.* Consistent with our results, reduced susceptibility was observed only in a limited subset of isolates, primarily associated with high-risk lineages carrying class D β-lactamases, underscoring the overall efficacy of cefiderocol against this species (Delgado-Valverde et al., 2020). Although ST2 strains showed higher MBC values than NST, these values, with one exception, remained within the susceptibility range, confirming cefiderocol’s broad-spectrum activity. Specifically, our study revealed that ST2 strains with dual positivity for *bla*_OXA-23_ and *bla*_OXA-82_ exhibited significantly higher cefiderocol MBC values compared to strains carrying *bla*_OXA-23_ and *bla*_OXA-66_, suggesting that these β-lactamase variants may differentially influence susceptibility to β-lactam antibiotics. Nevertheless, the consistent susceptibility of all isolates to cefiderocol supports its potential utility in managing infections caused by resistant *A. baumannii* strains. The addition of the class D β-lactamase inhibitor avibactam significantly reduced cefiderocol MICs in *bla*_OXA-23_-positive ST2 isolates, regardless of the presence of *bla*_OXA-66_ or *bla*_OXA-82_, indicating the predominant role of class D β-lactamases in cefiderocol activity. This finding aligns with studies showing that the combination of cefiderocol and avibactam can restore antibiotic susceptibility in MDR strains, suggesting that this combination could be an effective therapeutic strategy for treating resistant *A. baumannii* infections (Pasteran et al., 2024). Recent large-panel *in vitro* synergy testing reported marked potentiation of cefiderocol by β-lactam/β-lactamase inhibitor partners, particularly ceftazidime/avibactam and sulbactam/durlobactam, with consistent reductions in cefiderocol MICs across highly drug-resistant clinical isolates. These data support β-lactamase inhibitor–based combination strategies when cefiderocol activity is attenuated in resistant lineages (Fabrizio et al., 2025; Halim et al., 2025). Notably, two NST isolates (Ab09 and Ab17) lacked the *piuA* gene, which encodes a TonB-dependent receptor involved in iron uptake. However, these isolates did not exhibit increased resistance to cefiderocol, suggesting that the absence of *piuA* alone does not necessarily confer resistance in *A. baumannii*. This finding aligns with previous reports indicating that cefiderocol susceptibility is influenced by multiple factors, including alternative iron acquisition pathways, β-lactamase activity, and efflux pump expression (Stracquadanio et al., 2024). Further investigations are needed to elucidate the interplay between iron uptake mechanisms and cefiderocol resistance in *A. baumannii*.

These findings should be interpreted in light of important methodological limitations. First, siderophore production was assessed only by the CAS assay in culture supernatants, which provides an estimate of total iron-chelating activity but does not resolve individual siderophores or receptor-specific uptake pathways. Second, CAS analysis was not performed under biofilm conditions because iron-chelating activity measured in biofilm supernatants cannot be unambiguously attributed to biofilm-associated cells rather than detached/planktonic bacteria. Third, our genomic analysis identifies gene content but does not capture iron-regulated transcription or protein abundance. Therefore, although ST2 and NST isolates showed comparable iron acquisition gene content and CAS-detected siderophore activity under the tested conditions, we cannot exclude lineage-specific or growth-state-specific differences in the expression of iron uptake determinants that may influence cefiderocol susceptibility. Targeted RT-qPCR and/or proteomic analyses of key receptors such as *pirA, piuA*, and *tonB* under planktonic and biofilm conditions will be important in future studies.

Our findings highlight significant differences in biofilm-forming capacity between *A. baumannii* isolates from the ST2 and NST groups, indicating phenotypic heterogeneity that may be relevant to persistence in hospital settings. NST strains exhibited greater biomass production and higher biofilm cell density than ST2 strains, and microscopic analysis consistently showed thicker and more structured biofilms in the NST group. These differences may reflect underlying genotypic and phenotypic heterogeneity affecting biofilm architecture and bacterial aggregation, as previously reported among *A. baumannii* clones (Greene et al., 2016; Gedefie et al., 2023; Lichtenberg et al., 2023). Consistent with this framework, cefiderocol activity was reduced in the biofilm state, as reflected by significantly higher MBEC than MBC values and MBEC/MBC ratios ranging from 1 to 512. Notably, NST isolates, which displayed more structured biofilms in these assays, also exhibited significantly higher MBEC/MBC ratios than ST2 isolates. These observations support an association between biofilm-related phenotypes and reduced cefiderocol activity (Pybus et al., 2021), but do not establish a direct causal mechanism. As highlighted previously, the relationship between biofilm size and antimicrobial susceptibility is highly variable and influenced by multiple factors, including bacterial species, strain-specific traits, antibiotic class, and experimental design, with many studies reporting weak or inconsistent correlations and limited statistical support, often due to small sample sizes (Madduri et al., 2025). In this context, the present findings differ from those reported for *P. aeruginosa*, in which biofilm biomass did not show a robust association with cefiderocol activity, further underscoring species-specific and context-dependent biofilm–antibiotic interactions (Fabrizio et al., 2025).

Overall, these data support an association between biofilm-associated growth and reduced cefiderocol efficacy in this collection, while the underlying molecular determinants remain to be defined. From a translational perspective, these observations highlight biofilm growth as a clinically relevant factor that may complicate cefiderocol activity in *A. baumannii*.

## Data Availability

The datasets presented in this study can be found in online repositories. The data for this study have been deposited in the European Nucleotide Archive (ENA) at EMBL-EBI under accession number PRJEB85857.
