# Supplementary figures and images for: Clonal lineage and biofilm growth shape cefiderocol activity in Acinetobacter baumannii from oncology patients
Source: Front Cell Infect Microbiol. 2026 Apr 14;16:1788718. doi: 10.3389/fcimb.2026.1788718 (PMC13121156; doi:10.3389/fcimb.2026.1788718)

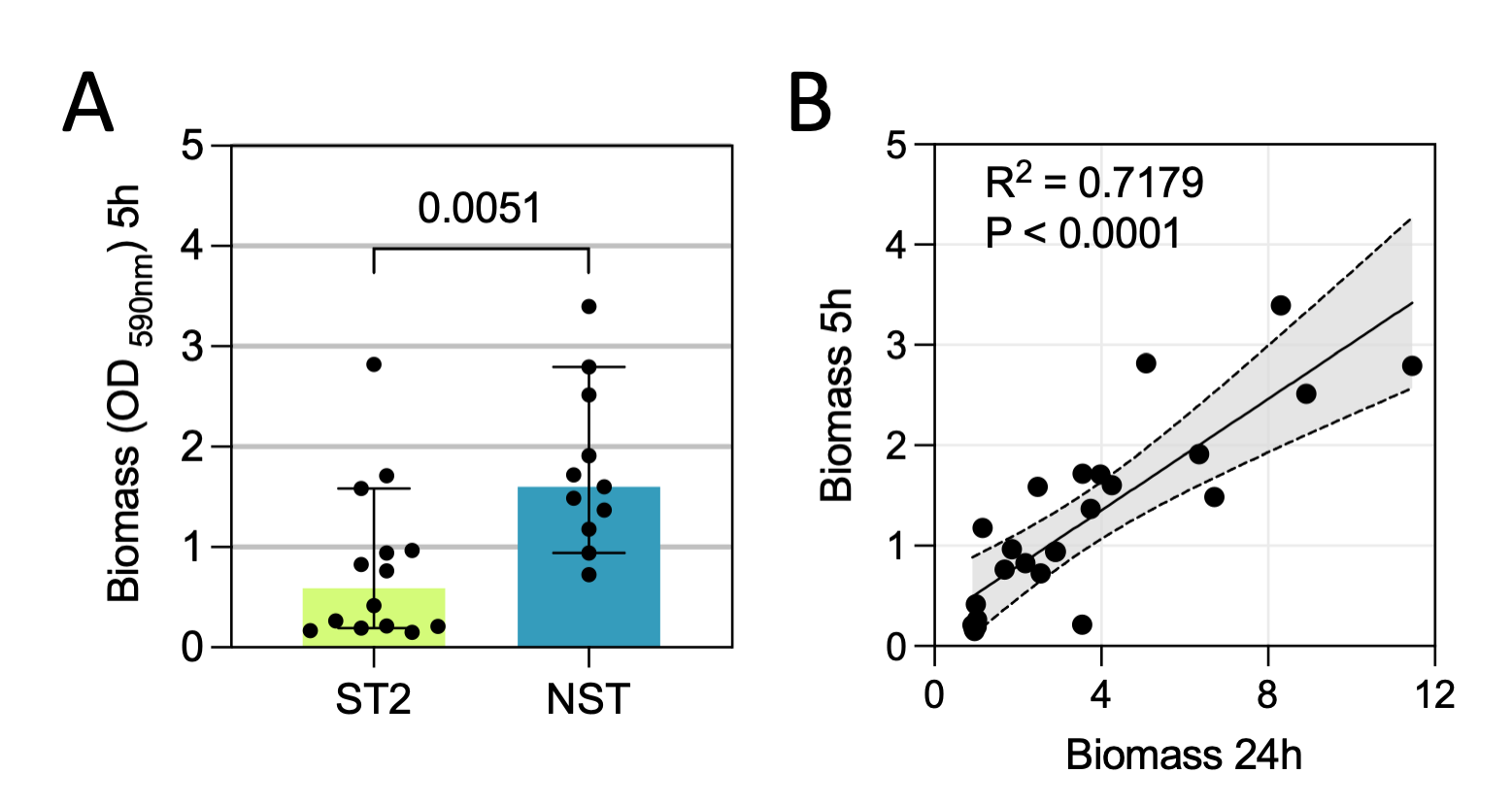

Supplement: Supplementary file 1 [file Image1.tiff]
